# Applications of artificial intelligence in early childhood health management: a systematic review from fetal to pediatric periods

**DOI:** 10.3389/fped.2025.1613150

**Published:** 2025-09-16

**Authors:** Qingsong Wang, Jun Yin, Xiaomeng Zhang, Huimin Ou, Fuyan Li, Yundong Zhang, Weiyi Wan, Caiyu Guo, Yongyu Cao, Tongyong Luo, Xianmin Wang

**Affiliations:** ^1^ Department of Pediatrics, West China Hospital Sichuan University Jintang Hospital. Jintang First People’s Hospital, Chengdu, Sichuan, China; ^2^ Department of Ultrasound, West China Hospital Sichuan University Jintang Hospital. Jintang First People’s Hospital, Chengdu, Sichuan, China; ^3^ Pediatric Cardiology Center, Sichuan Provincial Women’s and Children’s Hospital/The Affiliated Women’s and Children’s Hospital of Chengdu Medical College, Chengdu, Sichuan, China

**Keywords:** artificial intelligenc, machine learning, deep learning, fetal diseases, neonatal screening, child health, systematic review

## Abstract

**Background:**

The integration of artificial intelligence (AI) into early childhood health management has expanded rapidly, with applications spanning the fetal, neonatal, and pediatric periods. While numerous studies report promising results, a comprehensive synthesis of AI's performance, methodological quality, and translational readiness in child health is needed.

**Objectives:**

This systematic review aims to evaluate the current landscape of AI applications in fetal and pediatric care, assess their diagnostic accuracy and clinical utility, and identify key barriers to real-world implementation.

**Methods:**

A systematic literature search was conducted in PubMed, Scopus, and Web of Science for studies published between January 2021 and March 2025. Eligible studies involved AI-driven models for diagnosis, prediction, or decision support in individuals aged 0–18 years. Study selection followed the PRISMA 2020 guidelines. Data were extracted on application domain, AI methodology, performance metrics, validation strategy, and clinical integration level.

**Results:**

From 4,938 screened records, 133 studies were included. AI models demonstrated high performance in prenatal anomaly detection (mean AUC: 0.91–0.95), neonatal intensive care (e.g., sepsis prediction with sensitivity up to 89%), and pediatric genetic diagnosis (accuracy: 85%–93% using facial analysis). Deep learning enhanced consistency in fetal echocardiography and ultrasound interpretation. However, 76% of studies used single-center retrospective data, and only 21% reported external validation. Performance dropped by 15%–20% in cross-institutional settings. Fewer than 5% of models have been integrated into routine clinical workflows, with limited reporting on data privacy, algorithmic bias, and clinician trust.

**Conclusion:**

AI holds transformative potential across the pediatric continuum of care—from fetal screening to chronic disease management. However, most applications remain in the research phase, constrained by data heterogeneity, lack of prospective validation, and insufficient regulatory alignment. To advance clinical adoption, future efforts should focus on multicenter collaboration, standardized data sharing frameworks, explainable AI, and pediatric-specific regulatory pathways. This review provides a roadmap for clinicians, researchers, and policymakers to guide the responsible translation of AI in child health.

## 1 Introduction

Child health represents a cornerstone of global public health, with long-term implications for national human capital development and societal well-being. However, pediatric healthcare systems worldwide continue to face significant challenges. Approximately 2.3 million neonates die within the first month of life each year, the majority due to preterm birth complications, congenital anomalies, and perinatal conditions—many of which are preventable or treatable with timely intervention (1). Furthermore, the prevalence of chronic pediatric conditions such as congenital heart disease, neurodevelopmental disorders, and inborn errors of metabolism is rising, leading to prolonged functional impairment in affected individuals and imposing substantial economic and psychosocial burdens on families and healthcare systems. These disparities are exacerbated in resource-limited settings, where shortages of specialized pediatric personnel, inadequate diagnostic tools, and delays in early intervention contribute to persistent inequities in health outcomes. Consequently, enhancing the capacity for early detection, precise diagnosis, and individualized management of pediatric diseases has become a critical unmet need in global child health.

Recent advances in artificial intelligence (AI) have opened unprecedented opportunities to address these challenges (2). Machine learning (ML) techniques—including deep learning, natural language processing, and computer vision—have demonstrated exceptional performance in medical image analysis, physiological signal monitoring, genomic interpretation, and clinical decision support (3). In prenatal care, AI applications have enabled automated measurement of fetal biometric parameters, detection of fetal growth restriction (FGR), prediction of preterm birth, and high-accuracy diagnostic support across imaging modalities such as fetal ultrasound, Magnetic Resonance Imaging (MRI), and fetal electrocardiography. In postnatal pediatric care, AI models have been developed to analyze neonatal electroencephalography for seizure prediction, assess jaundice severity using smartphone-captured images, support staging of chronic kidney disease, and improve diagnostic accuracy for rare genetic disorders through facial phenotyping (e.g., Face2Gene) (4). These developments suggest that AI has the potential to shift clinical paradigms from passive documentation to proactive risk prediction and intelligent decision-making, thereby enhancing both the accessibility and precision of pediatric healthcare.

Despite this promise, the clinical translation of AI in fetal and pediatric care remains in its early stages and is hindered by multiple challenges. First, most existing models are trained on small, single-center datasets, lacking external validation and robust assessment of generalizability, resulting in a persistent “lab-to-clinic gap”. Second, pediatric data are inherently heterogeneous, developmentally dynamic, and highly sensitive, while high-quality, large-scale, and expertly annotated datasets specific to pediatric populations remain scarce—limiting model robustness and broad applicability. Third, the majority of current AI systems operate as “black boxes” with limited interpretability (Explainable AI, XAI), which undermines clinician trust and hinders clinical adoption. Additionally, pathways for clinical integration, ethical guidelines, regulatory frameworks, and cost-effectiveness evaluations for AI tools remain poorly defined, impeding their scalable deployment in real-world settings.

Given this context, a systematic evaluation of the current state of AI applications across the fetal-to-pediatric health continuum—assessing their evidence base, technological maturity, and translational potential—is both timely and essential. Recent research and empirical evidence on the implementation of AI in key domains—including fetal monitoring, neonatal intensive care, chronic disease prediction, and genetic disorder diagnosis—are synthesized. Technological bottlenecks and research gaps are identified, and future directions such as multimodal data integration, causal inference modeling, federated learning, and enhanced model interpretability are discussed. Table 1 summarizes the applications of AI across developmental stages, providing an overview of current use cases and clinical domains.

**Table 1:** AI applications in each stage.

Developmental Stage	Application Area	Specific Application	References
Fetal Period	Fetal	Prenatal screening for congenital abnormalities, optimization of prenatal diagnostic procedures	(5–7)
	Fetal Disease Diagnosis	Improvement of fetal ultrasound image quality, detection of spina bifida, prenatal diagnosis of congenital heart disease	(8–12)
	Fetal Echocardiography	Assistance in fetal growth monitoring, optimization of fetal ultrasound examinations	(13–18)
Neonatal Period	Neonatal Intensive Care	Multidimensional data analysis for early diagnosis, prediction and monitoring of neonatal diseases	(19–30)
Pediatric Period	Pediatric Clinical Practice	Disease diagnosis and prediction, screening and management of chronic diseases, optimization of surgical treatment	(31–48)
	Genetic Disease Diagnosis	AI-based genetic diagnostic programs, analysis of facial features, ocular structures, and skeletal imaging for genetic syndromes	(47, 49–72)

1. Fetal Period: 1.1 Fetal Medicine: AI technologies have significantly improved the accuracy of screening for congenital abnormalities and optimized prenatal diagnostic procedures.1.2 Fetal Disease Diagnosis: AI algorithms optimize ultrasound image quality, increase the detection rate of spina bifida, and achieve high accuracy in the prenatal diagnosis of congenital heart disease. 1.3 Fetal Echocardiography: AI assists in fetal growth monitoring and optimizes fetal ultrasound examinations, addressing issues such as high fetal activity and interobserver variability. 2. Neonatal Period: Neonatal Intensive Care: AI relies on big data analysis and machine learning algorithms to optimize early diagnosis and personalized treatment. Applications include sepsis risk prediction, mortality prediction, and monitoring of neonatal diseases. 3. Pediatric Period: 31 Pediatric Clinical Practice: AI is used in disease diagnosis and prediction, screening and management of chronic diseases, and optimization of surgical treatment. Applications include pediatric oncology, chronic disease management, and surgical navigation. 3.2 Genetic Disease Diagnosis: AI-based genetic diagnostic programs integrate clinical phenotype and genotype data to assist in the rapid identification of pathogenic mutations. Tools like Face2Gene, Eye2Gene, and Bone2Gene are used for the diagnosis of genetic syndromes.

## 2 Methods

### 2.1 Literature search strategy

Systematic literature searches were conducted in PubMed, Scopus, Web of Science, and IEEE Xplore to identify studies published between January 1, 2021, and March 15, 2025. The search strategy combined controlled vocabulary terms (e.g., MeSH terms in PubMed) with free-text keywords, adapted to the syntax of each database. Key concepts included artificial intelligence (e.g., “Artificial Intelligence”, “Machine Learning”, “Deep Learning”, “Neural Networks”) and pediatric populations (e.g., “Pediatrics”, “Neonatology”, “Fetus”, “Newborn Infant”, “Child Development”). Truncation symbols (*) were used to capture word variants (e.g., pediatr*, neonat*). Searches were limited to title and abstract fields where applicable.

In addition to electronic database searches, conference proceedings (*via* IEEE Xplore and ACM Digital Library) and preprint servers (arXiv and bioRxiv) were screened to capture emerging research. (Note: arXiv and bioRxiv are open-access preprint repositories for preliminary scientific reports prior to peer review.) The full PubMed search strategy is provided in Appendix 1. Study identification and screening were performed independently, with discrepancies resolved through consensus or arbitration by a third party.

### 2.2 Research questions

The review addresses the following research questions (RQs), formulated using the Population, Intervention, Comparator, Outcome, and Study Design (PICOS) framework:(RQ1) the applications of AI/ML in prenatal and perinatal care among pregnant women and fetuses, compared with standard care without AI support, examining the development and application of AI/ML models across any study design; (RQ2) the applications of AI/ML in neonatology among newborns and neonates, relative to standard care, focusing on diagnostic accuracy and clinical outcomes in prospective or retrospective cohort, cross-sectional, case–control, or randomised controlled trial (RCT) designs; (RQ3) the applications of AI/ML in paediatric disease management among children aged 0–18 years, against standard care, with outcomes of diagnostic accuracy and clinical effectiveness within the same study designs; (RQ4) the utilisation of AI/ML-based intelligent diagnostic technologies in paediatric populations (0–18 years), compared with conventional care, assessing diagnostic accuracy and clinical outcomes in prospective or retrospective cohort, cross-sectional, case–control, or RCT designs; (RQ5) the deployment of AI/ML-supported patient education and clinical decision support systems among patients, families, and healthcare providers, vs. standard care, evaluating knowledge retention and clinical utility within prospective or retrospective cohort, cross-sectional, case–control, or RCT designs; and (RQ6) the challenges and future directions for AI/ML in paediatric care, targeting healthcare providers and policymakers, contrasting current practices with proposed strategies to enhance implementation success and future potential, and including any study design reporting methodological or clinical limitations.

### 2.3 Inclusion and exclusion criteria

Peer-reviewed studies published in English between January 2021 and March 2025 were included if they reported diagnostic accuracy (e.g., sensitivity, specificity, area under the curve [AUC]) or clinical outcomes (e.g., mortality, morbidity, resource utilization) and employed prospective or retrospective cohort, cross-sectional, case–control, or randomized controlled trial (RCT) designs. Non-clinical studies, animal experiments, and studies based on unpublished or inaccessible data were excluded.

### 2.4 Study selection process

**Figure 1:**
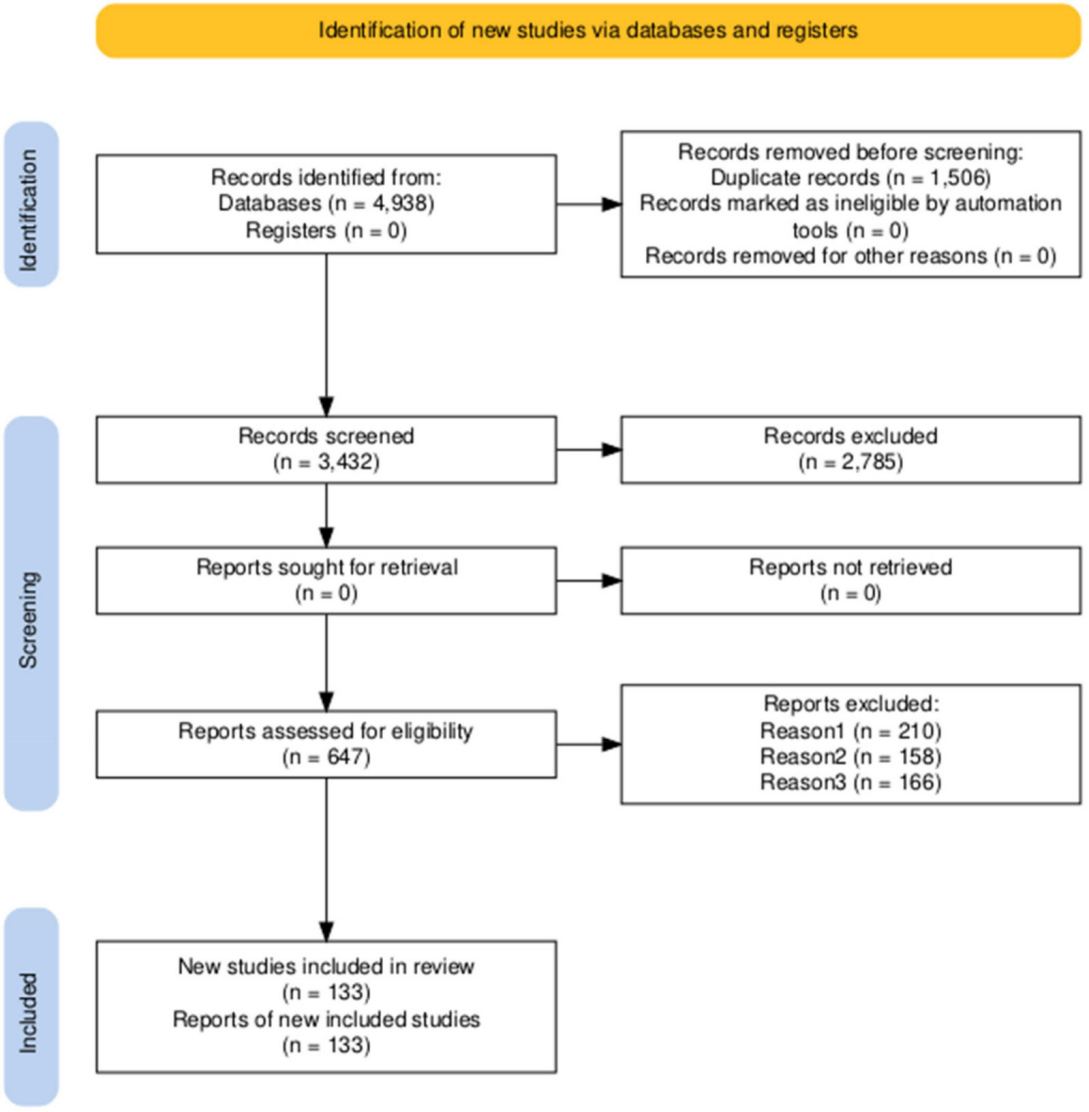
PRISMA flowchart of the included studies.

The study selection process followed the PRISMA 2020 guidelines and is summarized in Figure 1. An initial search yielded 4,938 records from electronic databases; no additional records were identified through other sources. After removal of 1,506 duplicates and exclusion of zero records flagged as ineligible by automated screening tools, 3,432 unique records remained for title and abstract screening. Of these, 2,785 were excluded as irrelevant, leaving 647 for full-text assessment. All 647 full-text articles were retrieved and evaluated against predefined inclusion criteria. A total of 514 studies were excluded: 210 were non-clinical, 158 involved animal experiments, and 166 were based on unpublished or inaccessible data. Ultimately, 133 studies met all inclusion criteria and were included in the final qualitative synthesis.

### 2.5 Quality assessment of included studies

A standardized quality assessment framework was applied to evaluate scientific validity and methodological rigor. The framework assessed study design, sample size, validation methods, performance metrics, and stage of clinical translation. Study designs were classified as prospective, retrospective, or multicenter, with multicenter prospective studies considered more robust. Sample sizes were categorized as large (≥1,000), medium (300–999), or small (<300). Validation methods were distinguished between internal and external validation, with greater weight assigned to studies using independent external datasets. All included studies reported at least one quantitative performance indicator, such as area under the receiver operating characteristic curve (AUC), sensitivity, or specificity. Studies were classified by stage of clinical translation: laboratory development, pilot application, or clinical implementation, with those demonstrating real-world deployment considered to have higher practical relevance.

The methodological quality of all 133 included studies was assessed using a five-domain framework outlined in Table 2. Studies were evaluated according to study design, sample size, validation strategy, reporting of performance metrics, and level of clinical translation, and assigned an overall quality rating: Grade A (≥12 points with external validation), Grade B (8–11 points), or Grade C (≤7 points).Of the 133 studies, 24 (18.0%) received Grade A, 71 (53.4%) Grade B, and 38 (28.6%) Grade C. High-quality examples include Pierucci et al. (4), Chen et al. (10), and Mallineni et al. (19) (all Grade A), while Alqahtani et al. (39) received Grade B. The predominance of single-center datasets and limited external validation highlights the need for more multicenter, prospectively validated pediatric AI research.

**Table 2:** QA quality-assessment criteria and evidence grading.

Domain	Criteria (from Section 2.4)	Weight	Grade
Study design	Multicentre prospective	3	A
Sample size	≥1,000	3	A
Validation method	External independent dataset	3	A
Performance metrics	≥3 metrics (AUC, sensitivity, specificity, etc.)	3	A
Clinical translation	Clinical deployment	3	A

Overall evidence grade A: total ≥ 12 points and external validation present B: 8–11 points C: ≤7 points.

### 2.6 Data extraction and categorization

Data extraction was conducted independently by two reviewers, with discrepancies resolved through consensus or third-party arbitration. Extracted data were categorized according to application domain. Methodological quality was assessed based on study design, sample size, validation approach, performance indicators, and translational stage. The level of evidence for each study was classified to reflect the reliability of findings and potential for real-world applicability. This approach supported reproducibility and transparency in synthesis.

## 3 Applications of artificial intelligence in prenatal and perinatal care

The integration of AI into prenatal and perinatal care has been associated with advances in risk prediction, imaging interpretation, and decision support. Evidence from recent studies can be organized into four thematic domains: (1) prediction of adverse pregnancy outcomes, (2) automation and standardization of ultrasound interpretation, (3) advanced imaging analysis in fetal MRI and echocardiography, and (4) augmentation of clinical expertise and reduction of diagnostic disparities. This framework reflects functional patterns in AI application and supports a structured synthesis of technological impact—highlighting roles in early detection, diagnostic consistency, and potential improvements in access to expert-level assessment. These applications address the first research question regarding the development and application of AI/ML models in prenatal and perinatal care.

### 3.1 Prediction of adverse pregnancy outcomes

Early identification of high-risk pregnancies is critical for timely intervention. AI models integrating multimodal data have demonstrated performance in predicting FGR and preterm birth. An AI system incorporating hemodynamic features for placental function assessment achieved a sensitivity of 89% for FGR detection, with clinical alerts generated 2–3 weeks earlier than conventional methods in one study (5). A support vector machine (SVM) model combining maternal physiological parameters and fetal monitoring data reported 91% accuracy and an AUC of 0.89 in predicting preterm birth (6). Multiparameter models analyzing fetal heart rate variability and uterine contraction frequency have identified preterm labor risk up to 48 h in advance (7). These findings suggest potential for earlier clinical actions, including corticosteroid administration or transfer to tertiary centers, although prospective validation in diverse populations remains limited.

### 3.2 Automation and standardization of ultrasound interpretation

Ultrasound remains the primary modality for fetal assessment, though diagnostic reliability may be affected by image noise, artifacts, and interobserver variability. AI algorithms have been applied to enhance image quality and automate biometric analysis. Deep learning techniques have reduced noise and artifacts, increasing the reported detection rate of spina bifida from 82%–94% in retrospective evaluations (8, 9). In fetal biometry, AI has improved measurement precision and reduced operator dependence (10). Deep learning models analyzing fetal ultrasound images achieved a classification accuracy of 97.2% in the prenatal detection of congenital heart disease, with lower error rates compared to manual measurements in controlled settings (11). Convolutional neural networks (CNNs) classified fetal renal pelvis dilation with 94% accuracy (95% CI: 93%–95%), and 51% of cases were correctly graded, indicating potential utility in borderline findings (18). These approaches may contribute to greater consistency in diagnostic workflows, particularly in complex or equivocal cases.

### 3.3 Advanced imaging analysis in fetal MRI and echocardiography

Beyond standard ultrasound, AI has been applied to enhance the utility of fetal MRI and echocardiography. In fetal MRI, AI algorithms have enabled organ segmentation, optimization of imaging sequences, and diagnostic support. Organ segmentation models achieved a Dice coefficient of 0.92 in identifying anatomical structures, suggesting improved efficiency in image interpretation (12). In fetal echocardiography, AI has supported image processing, biometric measurement, and anomaly detection, facilitating earlier identification of structural cardiac defects (13). Longitudinal monitoring of fetal growth through serial ultrasound analysis has also been demonstrated (14), with AI compensating for challenges such as fetal motion and maternal body habitus (15). In neuroanatomy, AI-based methods have enabled detailed assessment of brain development and detection of subtle abnormalities that may correlate with neurodevelopmental trajectories (16). Non-invasive fetal electrocardiography (fECG) integrated with AI improved the AUC of cardiac screening from 0.748 (resident)–0.890 (researcher) and 0.975 (expert), indicating performance gains across experience levels (17). The use of explainable AI (XAI) in cardiac screening, achieving an AUC of 0.975, may support transparency in decision-making (17).

### 3.4 Augmentation of clinical expertise and reduction of diagnostic disparities

AI-based systems have shown potential to improve diagnostic consistency in settings with limited access to specialized personnel. In low-resource environments, automatic fetal ultrasound screening systems increased the reported detection rate of congenital heart disease from 60%–89%, suggesting a narrowing of diagnostic performance gaps between high- and low-resource settings (73, 74). By standardizing image acquisition, view recognition, and anomaly detection, AI may assist clinicians across experience levels in achieving higher diagnostic accuracy. This domain highlights AI not only as a technical tool but as a potential contributor to more equitable access to prenatal screening across diverse populations, though real-world implementation evidence remains limited.

In summary, AI applications in prenatal and perinatal care span predictive modeling, imaging standardization, advanced diagnostics, and clinical support. From early risk prediction to image enhancement, advanced imaging analysis, and efforts to reduce diagnostic disparities, these technologies align with key clinical needs. While most models remain in the validation phase and face challenges in generalizability, regulatory approval, and integration into clinical workflows, the collective evidence indicates a trajectory toward earlier detection, reduced variability, and broader access to standardized fetal assessment. These developments suggest that AI is evolving from a supplementary tool to a potentially integral component of modern prenatal care systems.

## 4 Applications of artificial intelligence in neonatology

This section answers RQ2 by integrating evidence within four complementary functions: (1) real-time physiological monitoring and early warning, (2) neurodevelopmental surveillance and brain-injury prediction, (3) automated imaging interpretation, and (4) clinical decision support. These themes collectively illustrate the technical breadth of AI methodologies and their alignment with core neonatal care priorities—reducing diagnostic delays, minimising inter-observer variability, and enabling timely, individualised management for high-risk newborns.

### 4.1 Real-Time physiological monitoring and early warning

Detecting early signs of clinical deterioration in unstable neonates remains a critical challenge. Traditional monitoring systems generate frequent false alarms, contributing to alert fatigue among clinical staff. In contrast, AI models have been applied to high-frequency time-series data from electronic health records (EHRs) and bedside monitors to identify pre-symptomatic physiological patterns. Machine learning algorithms integrating heart rate variability, respiratory dynamics, and laboratory trends have demonstrated the ability to predict neonatal sepsis 6–12 h before clinical onset, with reported AUC values ranging from 0.85–0.93 in retrospective studies (18, 19). Predictive models for bronchopulmonary dysplasia (BPD) have utilized antenatal, postnatal, and ventilatory data to stratify risk, potentially guiding early pulmonary protective strategies (20, 21). These findings suggest that AI-based monitoring systems may support a shift toward earlier clinical recognition, although prospective validation in diverse Neonatal Intensive Care Unit (NICU) settings is still limited.

### 4.2 Neurodevelopmental surveillance and brain injury prediction

Timely identification of hypoxic-ischemic encephalopathy (HIE), seizures, and long-term neurodevelopmental impairment is essential for neuroprotective interventions. Given the limited availability of pediatric neurophysiologists, AI-based analysis of electroencephalography (EEG) — including amplitude-integrated EEG (aEEG) and raw EEG — has been explored as a scalable solution for continuous neurological monitoring. Deep learning models have achieved sensitivities exceeding 90% and specificities above 85% in automated seizure detection in preterm and term infants, enabling continuous monitoring even in resource-constrained environments (22, 23). Beyond seizure detection, AI frameworks such as dynamic functional connectome learning (DFC-Igloo) have been used to extract predictive biomarkers from resting-state functional Magnetic Resonance Imaging (fMRI), with reported correlations to motor and cognitive outcomes in preterm infants (24). Additionally, AI-powered cranial ultrasound analysis has enabled automated detection of intraventricular hemorrhage (IVH) and quantification of brain volume, providing objective and reproducible metrics for use in neuroprotection trials (25, 26). These approaches may contribute to more standardized and data-informed neurodevelopmental assessments.

### 4.3 Automated imaging interpretation

AI has been applied to standardize and accelerate diagnostic imaging in neonatal radiology and echocardiography. CNNs have been used for automated segmentation of cardiac structures, measurement of ventricular function, and detection of congenital heart defects in fetal and neonatal echocardiograms, with reported reductions in inter-observer variability (27, 28). In radiology, AI models have assisted in the interpretation of chest x-rays for conditions such as respiratory distress syndrome and pneumothorax, improving diagnostic speed and consistency (29). Computer vision algorithms have also been applied to video laryngoscopy, enabling real-time detection of glottic opening during intubation, which may support procedural accuracy (30). Furthermore, smartphone-based AI applications (e.g., BiliSG) have enabled non-invasive jaundice screening through digital color analysis, facilitating remote monitoring and reducing unnecessary blood sampling (31). These tools may extend diagnostic capabilities beyond tertiary centers, with potential implications for equitable access to neonatal care.

### 4.4 Clinical decision support

AI has increasingly been integrated into systems designed to support clinical decision-making and operational efficiency. AI-driven clinical decision support systems (CDSS) have been developed to integrate multimodal data for real-time optimization of ventilation settings, nutritional delivery, and antibiotic stewardship (75, 76). Predictive analytics have also been applied to discharge planning, identifying infants at elevated risk for readmission or developmental delay, thereby enabling targeted follow-up (77). At the systems level, AI models have been used to support NICU bed management, staffing forecasts, and patient flow optimization, with preliminary reports indicating improved resource utilization without compromising patient safety (78).

Synthesis across these four thematic areas indicates that AI applications in neonatology are evolving toward more data-driven, proactive, and individualized approaches to newborn care. The thematic clustering reflects a progression from diagnostic assistance (imaging, EEG) to risk prediction (sepsis, Bronchopulmonary Dysplasia (BPD), neurodevelopment) and intervention support (ventilation, intubation, nutrition). These developments align with the central aims of this review: AI-based tools have demonstrated high diagnostic accuracy (AUC > 0.85 in multiple studies), shown potential to reduce time-to-treatment and morbidity in observational settings, and exhibited increasing translational maturity. However, the majority of models remain in pilot or laboratory stages, with limited external validation. Key challenges include algorithmic bias, lack of interoperability with existing EHR systems, and insufficient evidence from multicenter prospective trials. Despite these limitations, the cumulative evidence suggests that AI may play an increasingly integral role in neonatal care, provided that future research prioritizes external validation, regulatory compliance, and equitable deployment across diverse healthcare settings.

## 5 Applications of artificial intelligence in pediatric disease management

This section responds to RQ3 by integrating evidence across two clinically focused domains: (1) the diagnosis and management of common paediatric diseases and (2) the diagnosis of genetic disorders. Within these domains, AI/ML applications consistently enhance diagnostic accuracy, refine risk stratification, and streamline clinical workflows—exemplified by earlier detection of congenital heart defects, improved prediction of sepsis onset, and accelerated identification of pathogenic variants—thereby enabling more individualised and timely care for children aged 0–18 years.

### 5.1 Diagnosis and management of common pediatric diseases

In pediatric oncology, ML models have been used to analyze high-dimensional clinical and biological data, identifying patterns associated with disease subtypes and treatment response. These models have demonstrated improved diagnostic precision and risk stratification in several pediatric cancers, supporting more individualized therapeutic approaches (79).

For the prediction of sepsis in children beyond the neonatal period, AI-driven models—primarily logistic regression and ensemble methods—have been applied to dynamic vital sign data, including heart rate variability and respiratory entropy. In a retrospective cohort study, such models predicted sepsis onset 6–8 h in advance with a reported sensitivity of 89% and specificity of 76% (31), suggesting potential for earlier clinical recognition, though prospective validation in real-world settings remains limited.

In pediatric malnutrition, ML algorithms have been developed to analyze multifactorial risk factors, including abnormal weight-for-age trajectories, to support early risk stratification. When integrated into web-based platforms, these models have enabled scalable screening tools for deployment in primary care and community health settings (32). Additionally, computer vision-based systems for infant posture tracking have been used to monitor subtle motor changes in real time, with one study reporting an accuracy exceeding 85% in detecting early signs of neurological impairment (33). These tools may enhance surveillance and support timely developmental interventions.

AI has also contributed to advances in the diagnosis of pediatric heart disease. Deep learning models trained on large ECG datasets have demonstrated the ability to detect early signs of congenital heart defects, such as atrial septal defects. In one validation study, the model increased diagnostic accuracy by 15% compared to conventional interpretation and reduced reported misdiagnosis rates to below 3% (34, 35). Furthermore, natural language processing (NLP) techniques have been applied to structured and unstructured echocardiographic reports to develop predictive models for spontaneous closure of perimembranous ventricular septal defects (PMVSD). One such model achieved an AUC of 0.92—18% higher than traditional parameter-based approaches—indicating improved predictive performance (36, 37). These findings suggest potential for earlier diagnosis and more personalized follow-up planning.

In the management of chronic pediatric conditions, AI-based tools have shown promise. Automated image analysis systems for pediatric diabetic retinopathy (DR) have been evaluated in clinical settings, demonstrating high sensitivity and specificity, with reported benefits in reducing clinician workload and improving access to screening in underserved populations (38). AI models have also been applied to assess environmental health risks in children. By integrating respiratory rate, environmental exposure data (e.g., PM2.5 concentration), and medication records, one predictive model achieved a reported accuracy of 91% in forecasting asthma exacerbations. In a pilot community health program, implementation of this model was associated with a 31% reduction in emergency department visits among asthmatic children, although the causal contribution of the AI component remains to be fully disentangled (39, 40).

In autism spectrum disorder (ASD) screening, AI-powered video analysis tools have been developed to detect behavioral markers—including facial expressions, eye contact, and limb movements—during brief (10-minute) interaction sessions. In a validation study of 1,200 children aged 2–5 years, the tool achieved a sensitivity of 94% and specificity of 89%, representing improvements of 20% and 30%, respectively, over the Modified Checklist for Autism in Toddlers (M-CHAT) (41). Another study reported that remote video-based behavioral analysis, combined with explainable AI (XAI) techniques, achieved a screening accuracy of 92% in infants as young as 18 months (42, 43). These approaches may support earlier identification, particularly in resource-limited settings.

In pediatric surgery, AI applications have been explored across preoperative planning, intraoperative guidance, and postoperative assessment. For laparoscopic cholecystectomy (LC), an AI system trained on liver CT imaging data has been used to identify fibrotic tissue and assist in surgical planning. In a clinical evaluation of 50 cases, the use of this system was associated with a reduction in biliary duct injury (BDI) rates from 8%–1.5%, and a decrease in planning time from 15–2 min, potentially reducing the need for intraoperative biopsies (44). In Hirschsprung's disease, an AI-assisted frozen section analysis system using transfer learning achieved a reported accuracy of 98.7% in ganglion cell detection (45). For retroperitoneal neuroblastoma, an AI-based 3D reconstruction model reduced surgical planning time by 40% in a single-center study (46).

Moreover, AI has shown strong performance in image-based differentiation of pediatric solid tumors. Multimodal image fusion techniques based on convolutional neural networks (CNNs) have demonstrated a sensitivity of 95% and specificity of 89% in distinguishing tumor types in retrospective analyses, highlighting the potential of AI in radiological and pathological interpretation (47).

### 5.2 Diagnosis of genetic diseases

AI has revolutionized the diagnosis of pediatric genetic diseases by enabling more accurate and efficient integration of phenotypic and genotypic data. Through advanced algorithms for phenotype-genotype association analysis and multimodal data fusion, AI tools are significantly reducing diagnostic delays and improving diagnostic yield in rare and complex genetic conditions.

AI-powered diagnostic platforms such as Dx29 integrate clinical phenotypes with genomic data to assist clinicians in rapidly identifying pathogenic variants in pediatric genetic disorders, including neurodevelopmental disorders and hearing impairments (48, 49). These systems streamline the diagnostic workflow by prioritizing candidate genes and reducing reliance on manual data interpretation. Similarly, AI tools like Franklin© AI reanalyze clinical genetic testing data to refine variant classification, thereby increasing diagnostic accuracy (50). In one large-scale study analyzing five years of pediatric genetic testing data, AI reanalysis identified 3,031 previously missed pathogenic variants, demonstrating substantial added value over conventional interpretation methods (51, 52).

AI is also being applied to congenital surgical conditions with genetic underpinnings. By analyzing genomic data, AI models can identify key disease-associated gene variants—such as those linked to Hirschsprung's disease or congenital diaphragmatic hernia—providing critical support for early diagnosis and surgical planning (53). Furthermore, AI-based risk prediction models integrate genetic and environmental factors to estimate the likelihood of pediatric-onset genetic disorders, such as hereditary cancer syndromes and neurodegenerative diseases, enabling earlier surveillance and intervention (54).

A major advancement in AI-assisted genetic diagnosis is the integration of electronic health records (EHRs), medical imaging, and genomic sequencing to support the identification of genetic syndromes. Deep learning algorithms can detect subtle morphological patterns in facial, ocular, and skeletal imaging, transforming phenotypic analysis into a quantitative, data-driven process (54–58). For example, Face2Gene—a widely used AI-based phenotypic analysis tool—analyzes facial photographs to generate a ranked list of up to 30 candidate syndromes, significantly accelerating the diagnostic process for rare diseases (59). It has demonstrated high performance in identifying conditions such as mucolipidosis type II (60) and has been validated in clinical settings in South Korea, showing strong diagnostic expansion potential (61). Studies confirm that Face2Gene's predictions for KBG syndrome and Kabuki syndrome are highly concordant with whole-exome sequencing results (62, 63). The tool also shows robust performance in diagnosing rare conditions such as Thrombocytopenia-Absent Radius (TAR) syndrome and Sotos syndrome, demonstrating its utility across diverse phenotypic spectra.

Beyond facial analysis, AI is being used to interpret ocular and skeletal imaging. Eye2Gene analyzes retinal and ocular images to detect features associated with inherited retinal diseases (IRD), improving variant interpretation and diagnostic confidence (64). Bone2Gene focuses on skeletal radiographs, using x-rays or computed tomography (CT) scans to identify dysmorphic features linked to genetic skeletal disorders, particularly those involving pathogenic variants in ANKRD11, and provides critical diagnostic clues in cases of skeletal dysplasia.

In genomic variant interpretation, AI systems are enhancing both speed and precision. The VAREANT platform improves variant detection and classification through three core modules: preprocessing, variant annotation, and AI/ML-driven data integration (65). The ClinGen Sequence Variant Interpretation (SVI) Working Group has endorsed the use of AI-based prediction tools in clinical variant classification, issuing updated guidelines to standardize their application (66). Deep learning models such as the Nucleic Transformer and Nucleotide Transformer have achieved state-of-the-art performance in DNA sequence classification and phenotype prediction (67–69). These pre-trained models are increasingly used to interpret non-coding region variants, a historically challenging area in clinical genomics (70).

To maximize accuracy, the integration of multiple AI models is recommended (71), and platforms like VarGuideAtlas are being developed to harmonize variant interpretation guidelines and support consensus-based classification (72). Such integrative approaches not only improve diagnostic sensitivity but also promote standardization across laboratories and healthcare systems.

In summary, AI technologies are transforming the landscape of pediatric genetic disease diagnosis. By leveraging facial, ocular, and skeletal imaging, as well as advanced genomic analysis, AI tools are shortening diagnostic odysseys, increasing diagnostic yield, and enabling earlier, more precise interventions. These advancements are laying the foundation for a new era of data-driven, personalized pediatric genetics.

## 6 Applications of intelligent diagnostic technologies

This section responds to RQ4 by evaluating AI/ML-based diagnostic technologies that enhance accuracy and clinical utility across pediatric medical imaging, pathology, and multi-omics analysis. These tools support automated image interpretation, anomaly detection, and integrated diagnostic reasoning, contributing to faster, more reliable, and standardized diagnostic processes in pediatric healthcare.

The increasing use of medical imaging in pediatric care has driven the integration of deep learning technologies across multiple modalities. AI-driven tools are now being applied to tasks including image classification, segmentation, prediction, and synthesis, with reported improvements in diagnostic consistency and workflow efficiency (47).

In ultrasound imaging, deep learning algorithms have demonstrated high performance in image quality assessment. One model achieved a reported recognition rate of 98.8% for normal pediatric sonographic images in a retrospective validation study, potentially reducing operator-dependent variability and enhancing reproducibility (20, 80). In radiology, AI-based bone age estimation from panoramic x-rays has shown a mean error of ±0.8 years—approximately half the error of traditional Greulich-Pyle method (±1.5 years)—supporting applications in growth assessment and orthodontic planning (81). A retrospective analysis of 3,000 pediatric dental radiographs found that an AI system for caries detection achieved 92% accuracy and 89% sensitivity, outperforming the average 78% accuracy of visual inspection by radiologists (37). More advanced architectures, such as the BAE-ViT visual transformer model, further improve performance by integrating clinical metadata (e.g., sex) with imaging data, representing a promising direction for multimodal diagnostic integration (82).

AI is also being explored in hybrid and cross-modality imaging. The generation of synthetic CT (sCT) from MRI data, when combined with PET, has been shown to reduce ionizing radiation exposure while maintaining diagnostic fidelity—offering a safer alternative for pediatric oncology and neuroimaging applications (83). In MRI, deep learning enables high-quality reconstruction from undersampled k-space data, potentially mitigating long-standing trade-offs between scan duration, spatial resolution, and signal-to-noise ratio (84). When integrated with quantitative MRI techniques, AI models can efficiently generate multi-contrast images, accelerating diagnosis in neurological disorders and supporting precision imaging workflows (85).

Beyond conventional imaging, emerging computational frameworks such as digital twins (DT) are being investigated for personalized pediatric care. By integrating physiological data, imaging phenotypes, and environmental factors, DT models aim to simulate disease progression and optimize treatment strategies. In pediatric neuro-oncology, the fusion of MRI with explainable AI (XAI) has enabled the development of adaptive clinical decision support systems that may improve both diagnostic interpretation and prognostic modeling (86, 87). Similarly, in chronic kidney disease (CKD), machine learning models leveraging multi-omics data have shown potential for early detection and risk stratification, potentially reducing unnecessary interventions (24).

AI is also accelerating biomarker discovery and enabling deeper biological insights. For example, AI-driven analysis of urinary proteomics and epigenetic profiles has identified non-invasive biomarker signatures for bladder cancer, suggesting potential to reduce reliance on invasive procedures (88). In hereditary kidney disease, integration of single-cell RNA sequencing with AI has elucidated molecular mechanisms of tubular injury in coenzyme Q10 deficiency nephropathy, revealing candidate therapeutic targets (89). In neuroblastoma, spatial transcriptomics combined with AI has identified a senescent-associated cancer-associated fibroblast (senes-CAF) subpopulation, offering new insights into tumor microenvironment modulation (90).

## 7 Applications in patient education and clinical decision support systems

This section responds to RQ5 by synthesizing AI/ML applications designed to improve knowledge delivery and clinical decision-making for patients, families, and healthcare providers. Through personalized education platforms and real-time decision support tools, AI helps bridge information gaps, enhance care coordination, and promote safer, more informed clinical practices.

In patient and medical education, AI-powered systems are being used to translate complex medical information into accessible formats. Text-to-video (T2V) generation models can produce dynamic visualizations of disease mechanisms or surgical procedures, facilitating both patient communication and medical training. In one reported application, such models reduced the production time of instructional videos—such as those demonstrating proper asthma inhaler technique—from 8 h–30 min, significantly improving content creation efficiency (91). Natural language processing (NLP)-based systems have also been developed to generate personalized health education materials tailored to a child's age, literacy level, and clinical context. In a pilot study involving families of pediatric kidney transplant recipients, the use of an AI-generated education tool was associated with a 35% improvement in caregiver knowledge retention, suggesting potential to enhance engagement and adherence (92). AI is also being explored in professional training; surveys of dental students' attitudes toward AI provide early insights for integrating intelligent technologies into clinical curricula (93).

In clinical operations and decision support, AI-driven systems have shown utility in high-acuity settings. Emergency department clinical decision support systems (ED-CDSSs) incorporating AI have been deployed for early sepsis detection and trauma severity grading. In a multicenter evaluation, such systems were associated with a 28% reduction in time to diagnosis, though challenges remain in processing unstructured clinical notes, with current models capable of analyzing only 32% of non-standardized text inputs—highlighting the need for more robust NLP integration (94). Augmented reality (AR)-assisted navigation systems have improved procedural accuracy in pediatric lumbar puncture, increasing first-attempt success rates from 68%–91% in a single-center study by providing real-time anatomical guidance (29).

Beyond bedside care, AI is being applied to hospital operations. One children's hospital implemented an AI-powered surgical scheduling system that analyzes historical procedure durations, resource utilization, and emergency priorities. This system increased operating room utilization from 68%–85%, reduced average patient wait times by 2.3 days, and was estimated to generate annual cost savings of $1.2 million (95). Such applications illustrate the potential of AI to enhance not only clinical outcomes but also healthcare efficiency and patient satisfaction.

## 8 Challenges and future development directions

This section responds to RQ6 by examining the methodological, technical, and translational barriers that hinder the clinical integration of AI/ML in pediatric healthcare. Key challenges include data heterogeneity, limited external validation, regulatory uncertainty, and poor alignment with clinical workflows. Addressing these barriers requires multicenter collaboration, standardized data practices, and human-centered design to enable equitable and sustainable implementation.

Despite the transformative potential of AI in pediatric healthcare, its translation from research to clinical practice faces multifaceted challenges that span ethical, methodological, and technological domains. At the ethical forefront, the use of children's sensitive health data raises urgent concerns regarding privacy, informed consent, and algorithmic equity. Only 19% of AI studies in pediatrics explicitly describe data anonymization procedures, and standardized protocols for obtaining meaningful consent—particularly from minors and their guardians—remain underdeveloped, increasing the risk of re-identification and misuse (96). Compounding this, training datasets often reflect racial, socioeconomic, and geographic biases, which can lead to degraded model performance in underrepresented populations, especially in low-income and resource-limited settings (87, 97). Without deliberate efforts to ensure representativeness and accessibility, AI risks exacerbating existing health disparities rather than mitigating them.

Methodologically, the field is hindered by significant heterogeneity in data and analytical approaches. A large proportion of studies rely on single-center, retrospective datasets, which are vulnerable to selection bias and lack external validation, limiting the generalizability of findings. Cross-institutional variations in data collection—such as imaging protocols, electronic health record (EHR) structures, and clinical workflows—can reduce model performance by 15%–20%, underscoring the critical need for standardized data curation and annotation frameworks (47, 98). Furthermore, the high cost of expert labeling and the scarcity of large, annotated pediatric datasets constrain the development of robust models, particularly for rare diseases. This is compounded by inconsistent model evaluation practices: while some studies report high accuracy, many fail to provide essential metrics such as sensitivity, specificity, or confidence intervals, making it difficult to compare performance across models or assess real-world utility.

The clinical adoption of AI is further impeded by the “black box” nature of many systems. Despite the introduction of visualization techniques to enhance interpretability, clinicians remain hesitant to trust AI-generated decisions without transparent, clinically meaningful explanations. Regulatory agencies increasingly demand rigorous documentation of model logic, uncertainty, and failure modes (99–101), highlighting the need for explainable AI (XAI) frameworks that align with clinical reasoning. For instance, deep learning models demonstrate high accuracy in detecting congenital heart disease from fetal ultrasound images but demand large datasets and substantial computational resources, whereas machine learning models offer faster inference and are better suited for real-time applications like neonatal sepsis prediction—yet without standardized benchmarks, such trade-offs remain poorly quantified.

Looking ahead, the future of pediatric AI lies in the integration of emerging technologies that enable more personalized, responsive, and secure care. Multimodal large language models (LLMs) hold promise for extracting and synthesizing complex information from unstructured EHRs, while digital twin technology could simulate individualized treatment responses by integrating longitudinal clinical, imaging, and genomic data. Edge computing devices offer the potential for real-time AI inference at the bedside—critical in neonatal and emergency settings—reducing latency and enhancing data privacy. Meanwhile, blockchain-based systems may provide a secure and auditable infrastructure for cross-institutional data sharing, fostering collaboration without compromising patient confidentiality (22, 47). However, the realization of this vision requires more than technological innovation; it demands systemic alignment through multicenter collaborations, standardized validation protocols, regulatory clarity, and human-centered design. Only by addressing these interconnected challenges can AI evolve from a collection of isolated tools into a trusted, equitable, and integral component of pediatric healthcare.

## 9 Practical implications for stakeholders

The findings of this review carry concrete and actionable implications for key stakeholders in pediatric healthcare, including clinicians, hospital administrators, policymakers, and technology developers. For clinicians, our synthesis highlights the growing maturity of AI tools in high-impact areas such as fetal anomaly detection, neonatal intensive care, and rare disease diagnosis—domains where early intervention is critical and human expertise is often stretched thin. Rather than viewing AI as a replacement, clinicians should position it as a cognitive augmentation tool that enhances diagnostic accuracy, reduces workload, and supports shared decision-making with families. However, this requires ongoing education in AI literacy, including understanding model limitations, interpreting uncertainty, and recognizing potential biases.

For hospital systems and healthcare administrators, the evidence underscores the need to invest in foundational infrastructure: standardized data pipelines, interoperable EHR systems, and secure computing environments (e.g., edge or federated learning platforms). The performance drop of 15%–20% when models are deployed across institutions is not merely a technical issue—it translates into real-world diagnostic delays and missed cases. Proactive investment in data harmonization and multicenter validation frameworks can mitigate this risk and accelerate the safe integration of AI into clinical workflows.

At the policy and regulatory level, our analysis reveals a critical gap: the absence of pediatric-specific AI governance standards. Unlike adult populations, children undergo rapid physiological and cognitive development, rendering adult-trained models potentially unsafe or inaccurate. Regulators must therefore establish developmental-stage-aware evaluation criteria, mandate transparency in training data demographics, and enforce strict privacy safeguards for lifelong pediatric data. Initiatives such as the FDA's Safer Technologies Program (STeP) offer a promising pathway, but they must be explicitly adapted for pediatric use cases.

Finally, for AI developers and researchers, this review calls for a paradigm shift—from building isolated “hero models” to designing clinically integrated, user-centered systems. Future efforts should prioritize external validation, real-world usability testing, and collaboration with frontline pediatric teams. The integration of multimodal LLMs, digital twins, and blockchain-based data sharing is not merely technological innovation; it is a systems-level opportunity to build proactive, personalized, and equitable child health ecosystems.

By aligning these stakeholder actions with the evidence synthesized in this review, the pediatric community can move beyond pilot studies and fragmented tools toward a future where AI is not an exception, but an expected, trusted, and life-enhancing component of routine care. The urgency is clear: every day of delay means missed opportunities for earlier diagnosis, more precise treatment, and better outcomes for the world's most vulnerable patients—children.

## 10 Conclusion

AI is profoundly reshaping the paradigm of pediatric medical practice. From real-time life monitoring in neonatal intensive care units (NICUs) to intelligent navigation in complex surgical procedures, from early warnings of fetal diseases to precise screening of pediatric diseases, the widespread application of AI technologies in the pediatric field demonstrates strong clinical potential. AI technologies not only improve the accuracy of diagnosis and treatment but also optimize the allocation and utilization of medical resources. However, continuous technological innovation needs to be synchronized with the standardization of methodologies, the improvement of ethical frameworks, and the construction of interdisciplinary collaboration systems. Future research should focus on standardized validation methods and multicenter studies to ensure the robustness and universality of AI applications. At the same time, addressing ethical and privacy issues is crucial for the widespread adoption of AI in pediatric healthcare. Looking ahead to the next 5–10 years, with the continuous expansion of pediatric-specific datasets and the application of cutting-edge technologies such as causal inference, innovations and breakthroughs in the pediatric field will bring new hope to the cause of children's health. The future development of AI technologies in the pediatric field is expected to further development of AI technologies in the pediatric field, which can provide more precise and efficient support for the health and well-being of children. These directions not only fully demonstrate the multi-level application potential of AI technologies from basic research to clinical implementation but also point the way for the future development of pediatric healthcare.

## Data Availability

The original contributions presented in the study are included in the article/supplementary material, further inquiries can be directed to the corresponding authors.
